# Independent evolution of the core and accessory gene sets in the genus *Neisseria*: insights gained from the genome of *Neisseria lactamica *isolate 020-06

**DOI:** 10.1186/1471-2164-11-652

**Published:** 2010-11-23

**Authors:** Julia S Bennett, Stephen D Bentley, Georgios S Vernikos, Michael A Quail, Inna Cherevach, Brian White, Julian Parkhill, Martin CJ Maiden

**Affiliations:** 1 Department of Zoology, University of Oxford, South Parks Road, Oxford, OX1 3PS, UK; 2 The Wellcome Trust Sanger Institute, Wellcome Trust Genome Campus, Hinxton, CB10 1SA, UK

## Abstract

**Background:**

The genus *Neisseria *contains two important yet very different pathogens, *N. meningitidis *and *N. gonorrhoeae*, in addition to non-pathogenic species, of which *N. lactamica *is the best characterized. Genomic comparisons of these three bacteria will provide insights into the mechanisms and evolution of pathogenesis in this group of organisms, which are applicable to understanding these processes more generally.

**Results:**

Non-pathogenic *N. lactamica *exhibits very similar population structure and levels of diversity to the meningococcus, whilst gonococci are essentially recent descendents of a single clone. All three species share a common core gene set estimated to comprise around 1190 CDSs, corresponding to about 60% of the genome. However, some of the nucleotide sequence diversity within this core genome is particular to each group, indicating that cross-species recombination is rare in this shared core gene set. Other than the meningococcal *cps *region, which encodes the polysaccharide capsule, relatively few members of the large accessory gene pool are exclusive to one species group, and cross-species recombination within this accessory genome is frequent.

**Conclusion:**

The three *Neisseria *species groups represent coherent biological and genetic groupings which appear to be maintained by low rates of inter-species horizontal genetic exchange within the core genome. There is extensive evidence for exchange among positively selected genes and the accessory genome and some evidence of hitch-hiking of housekeeping genes with other loci. It is not possible to define a 'pathogenome' for this group of organisms and the disease causing phenotypes are therefore likely to be complex, polygenic, and different among the various disease-associated phenotypes observed.

## Background

Comparison of the genomes of related bacteria that exhibit distinct pathogenic phenotypes can identify the genetic traits required for invasion and elucidate key steps in the evolution of virulence. The genus *Neisseria*, which comprises Gram negative oxidase positive diplococci that colonise the mucosa of humans and animals, provides an excellent model for this type of study as it includes species that are never or rarely pathogenic and two human pathogens of global significance, *Neisseria meningitidis *(the meningococcus) and *Neisseria gonorrhoeae *(the gonococcus) [1]. *Neisseria lactamica *is closely related to the pathogenic *Neisseria *[2,3] and, like them, is only ever isolated from humans; consequently, a number of studies have been undertaken to compare the non-pathogen *N. lactamica *with meningococci and gonococci in the hope of identifying key genetic determinants of meningococcal or gonococcal disease [4-13].

Phenotypically, the gonococcus is the most divergent of the three organisms as it colonises the urogenital tract and can be considered to be an obligate pathogen of the mucosal surface that occasionally causes disseminated infection [14]. The meningococcus and *N. lactamica *are more similar in their life histories: both are obligate commensal inhabitants of the human nasopharynx that establish long-term normally asymptomatic colonisation. Carriage of *N. lactamica *is high in infants and young children and declines as the age of the human host population rises. The converse is true for the meningococcus, the carriage prevalence of which is low in infants and young children but rises with host age, generally reaching its highest in adolescents and young adults [15,16]. Unlike *N. lactamica*, which is only anecdotally associated with invasive disease [17-19], the meningococcus can be a dangerous pathogen occasionally invading the nasal mucosa to cause septicaemia and meningitis [20]. Although devastating for the patient, neither of these syndromes is of any benefit to the meningococcus itself as they do not normally lead to onward transmission of the bacterium, which is therefore best categorised as an 'accidental pathogen' [21]. Nonetheless, meningococcal disease is a global phenomenon which, in some settings, occurs in large outbreaks [22]. The idea that the colonisation of children with *N. lactamica *plays a role in the development of immunity to the meningococcus [23-25] has further stimulated comparative investigations of these two organisms and anti-meningococcal vaccines based *N. lactamica *have been proposed at various times [26,27].

The meningococcus, gonococcus, and *N. lactamica *are closely related at the genetic level [2] and appear to have recently descended from the same ancestral population. Multilocus studies have indicated that these are maintained as separate populations by the absence or low frequency of genetic exchange among them, although rates of recombination within each of the microbiological species groups are high [3] and some genetic sequences are shared among species groups. The low genetic diversity observed at seven housekeeping genes of the gonococcus is consistent with this organism having evolved from a single clone that changed niche from the nasopharyngeal to the urogenital tract [28]. *N. lactamica *and meningococcal populations, on the other hand, are more diverse and both populations consists of a number of clonal complexes, each comprising related genotypes [29,30]. In the case of the meningococcus, some of these, the so-called hyperinvasive lineages, are particularly associated with invasive disease [31]. Knowledge of the population structures of these organisms has been used to design studies of the genetic elements that are involved in virulence [4,8,13,32], but to date a pathogenic genotype has yet to be determined and it seems probable that the invasive phenotype of the hyperinvasive genotypes is complex and polygenic. Here we examine the genealogical relationships among the three species, describe the determination and annotation of the first complete genome sequence of *N. lactamica *(isolate 020-06) and discuss the insights that this provides into the evolution of the pathogenic *Neisseria*.

## Methods

### Genealogical relationships among species

Relationships among *N. meningitidis*, *N. lactamica *and *N. gonorrhoeae *were investigated by an analysis of nucleotide sequences from 19 housekeeping gene loci. The loci included were those used for *Neisseria *MLST (*abcZ*, *adk*, *aroE*, *fumC*, *gdh*, *pdhC*, and *pgm*) [29,33] supplemented with 12 additional loci (*aspA*, *carB*, *dhpS*, *glnA*, *gpm*, *pilA*, *pip*, *ppk*, *pykA*, *rpiA*, *serC*, *talA*) with alleles generated as described previously [34]. The analysis included data from 52 *N. lactamica *isolates, comprising 46 unique seven locus sequence types (STs) sampled from a population of 271 isolates used to analyse diversity in *N. lactamica *[30], 20 isolates representing 20 unique STs (one ST from each clonal complex and two STs not belonging to clonal complexes) from the 107 *N. meningitidis *isolates originally used to define MLST [29], and subsequently analysed at additional loci [34], and seven complete *Neisseria *genomes downloaded from publicly available databases. These genomes include: meningococcal isolates Z2491 (serogroup A, ST-4 complex) [35], MC58 (serogroup B, ST-32 complex) [36], FAM18 (serogroup C, ST-11 complex) [37], 053442 (serogroup C, ST-4821 complex) [38], α14 [13], and gonococcal isolates FA1090 (The University of Oklahoma, U.S.A), and NCCP11945 [39]. All isolates used (Additional file 1) have been deposited in the *Neisseria *PubMLST database: http://pubmlst.org/neisseria/, which is searchable by publication record.

Genealogies were constructed with CLONALFRAME version 1.1 [34,40] using 50000 burn-in iterations and 50000 Monte-Carlo Markov Chain iterations. The results from eight runs were combined to achieve maximum robustness and the 75% consensus tree was viewed using MEGA version 4.0 [41]. To add support to the species-specific clustering, neighbour-joining trees were drawn from the same data using MEGA version 4.0 and Split Decomposition was carried out using SplitsTree version 4.10 [42]. MEGA version 4.0 was used to determine p distances.

### Determination of the complete genome sequence of *N. lactamica *020-06

The genome was sequenced to an approximately 10-fold shotgun sequence, totaling 42232 end sequences, from pUC19 (with insert sizes of 1.4-2 kb; 2-2.8 kb and 3-3.3 kb respectively. 2.8-3.3 kb), and pMAQ1Sac_BstXI (with insert sizes of 5.5 kb-6; 9-10 and 10-12 kb respectively) genomic shotgun libraries using big-dye terminator chemistry on ABI3730 automated sequencers. End sequences from large insert BAC libraries in pBACe3.6_BamHI with an insert size 15-18 kb were used as a scaffold. This generated an approximate 1-fold coverage from 4670 end sequences). All repeat regions and gaps were bridged by read-pairs or end-sequenced polymerase chain reaction (PCR) products again sequenced with big dye terminator chemistry on ABI3730 capillary sequencers. The sequence was manipulated to the 'Finished' standard [43].

### Annotation and genome comparison

An automatic gene prediction program (Glimmer3) [44] was used to identify coding regions. Putative orthologues were identified by reciprocal-best-match FASTA searches between *N. lactamica *020-06 and meningococcal strain FAM18 amino acid sequences with cut-offs of 80% sequence length and 30% identity. The complete genome was annotated manually using the genome viewer Artemis [45]. The *N. lactamica *genome sequence was compared to genomes from *N. meningitidis *(Z2491, MC58, FAM18, 053442 and α14) and *N. gonorrhoeae *(FA1090, NCCP11945) using the Artemis comparison tool (ACT) [46]. The Dot plot figure was generated using MUMmer version 3.22 [47] and indicates matching sequences, with codirectional and reversed regions of synteny shown in red and blue, respectively. The figure is a plot for multiple query sequences (other *Neisseria*) and one reference sequence (*N. lactamica*) where each reference/query comparison gives its dotplot. The genome sequences were aligned to start/finish at the origin of replication. The comparative analysis of the core gene dataset was carried out using an in-house pipeline of all-against-all best reciprocal FASTA searches that outputs the respective percentage (pair-wise) identity. The analysis and annotation of repeat families in *N. lactamica*, was carried out implementing a hidden Markov model based methodology, as previously described [37].

## Results and Discussion

### Genealogical relationships of *N. lactamica*, *N. meningitidis*, and *N. gonorrheoae*

**Figure 1:**
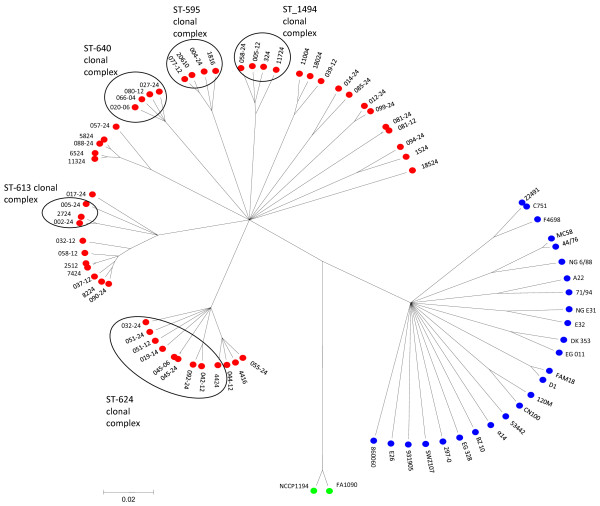
**Genealogical relationships of *Neisseria *isolates inferred from 19 loci**. CLONALFRAME trees were drawn from the sequences from 19 housekeeping gene fragments from 79 isolates. These include the eight complete *Neisseria *genomes, 20 isolates representing 20 unique seven locus STs (one ST from each clonal complex and two STs not belonging to clonal complexes) from the meningococcal collection used to validate the MLST scheme [29], and 51 *N. lactamica *isolates, comprising 46 unique seven locus STs sampled from a population used to analyse diversity in *N. lactamica *[30]. *N. lactamica *clonal complexes are circled. *N. lactamica *(Nla) = red, *N. meningitidis *(Nme) = blue, *N. gonorrhoeae *(Ngo) = green.

The nucleotide sequences of 19 housekeeping genes from 52 *N. lactamica*, 25 meningococcal and two gonococcal isolates were used to generate a genealogy with the CLONALFRAME algorithm [40]. ClonalFrame uses a statistical algorithm that infers clonal relationships while taking into account the effect of homologous recombination. This algorithm clustered the isolates into three groups, each corresponding to one of the microbiological species (Figure 1). These groups were also evident from other clustering algorithms, including neighbour joining trees and split decomposition (Additional file 2). Neighbour joining trees for each of the individual loci mostly replicated the same groups with some inconsistencies (Additional files 3, 4 and 5); for example, the genealogy for the *dhps *locus did not show species-specific clustering for *N. meningitidis *and *N. lactamica*, which might suggest horizontal genetic exchange between species. As *dhps *encodes dihydropteroate synthase, and sulphonamide resistance is mediated by altered forms of this enzyme [48], positive selection may promote recombination at this locus. However, no *dhps *alleles were shared among the three species.

The nucleotide sequence diversity for the 19 loci among the species groups as assessed by pairwise p-distances was similar, ranging from a p-distance of 0.053 between the meningococci and the gonococci to a p-distance of 0.073 between *N. lactamica *and *N. meningitidis*. The diversity within each of the groups, measured by the same means, was also similar among the *N. lactamica *isolates (average p-distance of 0.026) and the meningococcal isolates (average p-distance of 0.032) and appreciably lower between the two gonococcal isolates (p-distance of 0.06). These data support the conclusions reached from seven-locus multilocus sequence typing (MLST) and multilocus enzyme electrophoresis studies that the three microbiological species represent valid functional and taxonomic groups [3,28] notwithstanding their close genetic similarity [2], which is consistent with recent divergence.

For the isolates included in this analysis, the number of fixed nucleotide differences greatly exceeded the number of shared polymorphisms in comparisons between the gonococci and meningococci (147 fixed differences, 33 shared polymorphisms) and the gonococci and *N. lactamica *isolates (316 fixed differences, 11 shared polymorphisms). This contrasted with comparisons of the meningococcal and *N. lactamica *isolates, where the number of fixed differences (118) was much smaller than the number of shared polymorphisms (435). Taken together these data indicate that all three species groups share an ancestral population, with the gonococcus likely to be descended from a single member of that population. *N. gonorrhoeae *does not exchange genetic material with other *Neisseria *frequently but, unlike other pathogens that have emerged by a similar process [49,50], genetic material is regularly exchanged among gonococci such that their populations do not have a clonal structure [51,52]. Present day meningococcal and *N. lactamica *populations, on the other hand, have diverged from the ancestral population without undergoing a severe bottleneck and now represent distinct species groups, with the nucleotide sequence diversity in their housekeeping genes exhibiting extensive shared ancestry.

In the current analysis there were only two cases of alleles shared among the different species groups, both at the *pykA *locus. The *pykA-7 *allele was present in 5 meningococcal isolates and one *N. lactamica *isolate and the *pyKA-24 *allele was present in one meningococcal isolate and one *N. lactamica *isolate. In the *N. lactamica *020-06 genome this locus is immediately adjacent to an orthologue of gene NMB0088 in the meningococcal MC 58 genome, which encodes a putative immunogenic outer membrane protein orthologous to the FadL potential vaccine component of *Escherichia coli *[53]. This observation complements previously published evidence for the hitch-hiking of housekeeping alleles with antigen alleles promoting genetic exchange among meningococci and *N. lactamica *[54], although such hybrids as do occasionally arise appear to be less fit and are purged by purifying selection [55]. In conclusion, while genetic exchange between meningococci and *N. lactamica *has been reported for a number of genes under positive selection [55,56], the two groups remain distinct as a result of limited genetic exchange among housekeeping genes [3], which is consistent with the species status of these organisms. Frequent horizontal genetic exchange among groups would prevent speciation, or if it occurs after the speciation event, would lead to a 'despeciation' process, as has been proposed recently for the gastric pathogens *Campylobacter jejuni *and *Campylobacter coli *[57]. There is no convincing evidence of such a process occurring among the *Neisseria*.

As with meningococci, *N. lactamica *isolates are currently assigned to clonal complexes on the basis of their seven locus sequence types [30]http://neisseria.org/nm/typing/mlst/. These clonal complexes comprise a central genotype ST and STs that share identical alleles for at least four of the seven MLST loci in the central genotype. At the time of writing, six *N. lactamica *clonal complexes had been identified, each named after the central genotype of the complex (the ST-595, ST-613, ST-624, ST-640, ST-1494 and ST-1540 clonal complexes), five of which were represented in the genealogical analysis reported here. Members of the same clonal complexes were consistently clustered together in the 19 locus genealogy, indicating the general robustness of the assignment of clonal complexes on the basis of seven loci. The genealogy did, however, suggest that some of the genealogical groups were larger than the currently defined clonal complexes and also indicated the existence of several other clonal complexes, currently represented by one or a few sequence types (Figure 1). The star phylogeny of *N. lactamica *is redolent of that of the meningococcus [34] and consistent with both species exhibiting a clonal complex population structure, comprising groups of related isolates that cannot be genealogically linked to each other as a consequence of high rates of intra-species horizontal genetic exchange [33,34,58].

On the basis of these analyses there was no *a priori *reason to choose a member of one particular clonal complex over any other for genome sequencing, although it was considered desirable to choose a recent bacterial isolate that had not been maintained in the laboratory for long periods of time and one that represented a common and widespread genotype. For these reasons, isolate 020-06, obtained from a six-week-old child from the UK in 1997 was chosen as the most appropriate candidate for genome sequencing. This isolate has ST-640, the central genotype of a clonal complex from which members have been isolated in the UK [30] and four other countries (unpublished data). The MLST profiles for these isolates can be viewed at: http://pubmlst.org/neisseria/[59].

## Comparison of the genome sequence of *N. lactamica *isolate 020-06 with other *Neisseria *genomes

### General features and gene order

The complete genome sequence of isolate 020-06 was determined by capillary-based whole-genome shotgun with standard finishing procedures. It has been deposited in GenBank with the accession number FN995097. The genome comprised a circular chromosome of 2,217,455 bp in length, with a GC content of 52.27%. These features were very similar to those of the published meningococcal and gonococcal genomes which ranged from 2,145,295 bp to 2,272,351 bp in length, with a GC contents ranging from 51.53% - 52.68%. The numbers and distribution of DNA uptake sequences (DUS, 2244), Correia elements (138), and dRS3 repeats (297) were broadly similar to those seen in the other published *Neisseria *genome sequences and have been used to suggest that genetic exchange represents a conservative rather than diversifying force in the *Neisseria *[60]. The number of predicted coding sequences (CDSs) identified in the genome of 020-06, at 2018, was also similar to the number identified in the other genomes, which ranged between 1976 and 2662 CDSs, although this number is not necessarily directly comparable owing to differences in the annotations of the published and unpublished genome sequences [13,35-39] (Table 1). In addition, a plasmid of 3,151 bp in length was present, similar to *N. lactamica *plasmid pNL18.2 [61]. Plasmids are relatively common in *N. lactamica *and the gonococcus, although rarely seen in meningococci [61].

**Table 1:** General features of eight Neisseria genomes

	020-06	Z2491	MC58	FAM18	053442	α 14	FA1090	NCCP11945
Species	Nla	Nme	Nme	Nme	Nme	Nme	Ngo	Ngo
Disease or carriage	Carriage	Disease	Disease	Disease	Disease	Carriage	Disease	Disease
Serogroup	N/A	A	B	C	C	Capsule null	N/A	N/A
MLST sequence type	ST-640	ST-4	ST-74	ST-11	ST-4821	ST-53	ST-1899	ST-1901
MLST clonal complex	ST-640	ST-4	ST-32	ST-11	ST-4821	ST-53	N/A	N/A
Genome size (bp)	2217455	2184406	2272351	2194961	2153416	2145295	2153922	2232025
G + C content (%)	52.27	51.81	51.53	51.62	51.7	52.0	52.68	52.4
Putative No. of CDSs	2018	1999*	2024*	1976*	2051	1987	2002	2662

* No. of CDSs as per Bentley et al [37].

Nme = *N. meningitidis*, Nla = *N. lactamica*, Ngo = *N. gonorrhoeae*

N/A, not applicable

**Figure 2:**
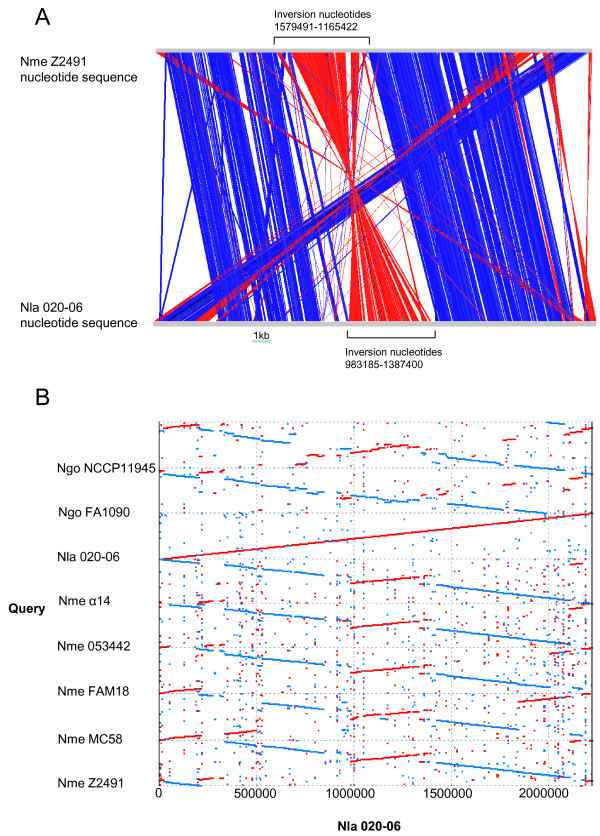
**Comparisons of genome organization**. **2A. A comparison between the genomes of *N. meningitis *Z2491 and *N. lactamica *020-06 using ACT **[46]. The apparent re-arrangement at the start of the Z2491 genome with respect to *N. lactamica *020-06 was because the start of the Z2491 genome was not assigned at the origin of replication. **2B. **A dot plot comparing the genome sequence of *N. lactamica *020-06 with the seven other complete *Neisseria *genomes.

Whole genome comparisons of gene order showed that, with the exception of an inversion of 404215 bp around the terminus of genome replication, the genome sequence of *N. lactamica *020-06 was essentially co-linear with that of meningococcal isolate Z2491 (Figure 2A). The larger inversion was also evident in comparisons of the *N. lactamica *genome with the genome sequences of the remaining meningococcal genomes examined, those of isolates MC58, FAM18, α14, and 053442, one of the two gonococcal isolates, NCCP11945, but not the genome of isolate FA1090. Other than these changes in gene order, all of which maintained features such as GC skew (Additional file 6), there were no consistent changes in co-linearity among any of the *Neisseria *genomes examined (Figure 2B). Although the total number of completely assembled genomes available remains small, these comparisons suggest that there is a consensus genome order, described previously for *N. meningitidis *[37], for all three *Neisseria *species examined here. This is essentially the gene order seen in meningococcal isolates Z2491, α14, 053442, and FAM18. Most of the large-scale departures from this consensus are present in only one meningococcal genome examined so far and may be particular to that isolate alone or that isolate and its close relatives. Such rearrangements, frequently observed in bacteria [62] may well be biologically neutral or artifacts of growth in the disease state [63] or pure culture. One exception to this was the large rearrangement around the terminus that is present in the *N. lactamica *isolate and one gonococcal isolate (FA1090). The very close relationship of all gonococci (Figure 1), suggests that this inversion may have occurred more than once in the history of the genus, but more genome data are required to definitively answer this question. There is consequently no evidence to suggest that gross genome rearrangements have played a role in the emergence of pathogenicity in the *Neisseria *as there is no consistent pattern of genomic rearrangements among non-pathogenic and pathogenic *Neisseria*, as have been observed in some other groups containing bacterial pathogens [64], although a role of such rearrangements in the emergence of clonal complexes cannot be excluded on the basis of information currently available.

### Gene content and core genome

**Figure 3:**
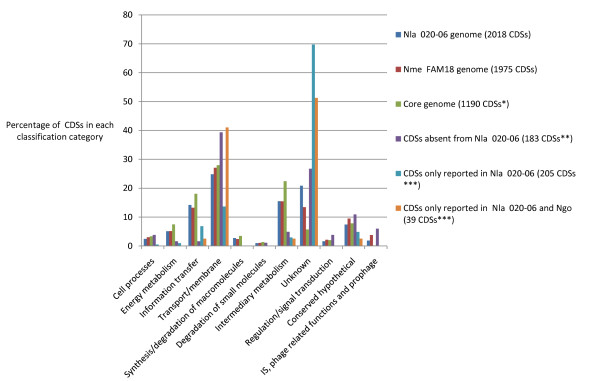
**Percentage of CDSs in each classification category**. * Data produced by comparing the genome of *N. meningitidis *Z2491 with the genomes of *N. lactamica *020-06, *N. meningitidis *genomes MC58, FAM18, 053442, α14, and N. gonorrhoeae genomes FA1090 and NCCP11945. ** Data produced using the annotation from FAM18 and by comparing the genomes of *N. lactamica *020-06, *N. meningitidis *genomes Z2491, MC58, FAM18, and *N. gonorrhoeae *genome FA1090. *** NCBI BLAST search 16-11-09. Nme = *N. meningitidis*, Nla = *N. lactamica*, Ngo = *N. gonorrhoeae*

Of the 2018 *N. lactamica *CDSs identified, 1447 (72%) were assigned to functional categories. Of the remaining CDSs, 421 (21%) were not assignable to a known function and 150 (7%) were categorized as conserved hypothetical proteins. Comparisons with the genomes of *N. meningitidis *Z2491, MC58, FAM18, 053442, α14, and *N. gonorrhoeae *genomes FA1090 and NCCP11945 showed a similar distribution of functions (Figure 3). Changes in gene content among the different genome sequences were confined to CDSs classified as involved in transport and metabolism. These were relatively overrepresented among those CDSs present in other *Neisseria*, but absent in *N. lactamica*. They were also underrepresented among those only present in the *N. lactamica *genome. CDSs of unknown function were overrepresented in the *N. lactamica *genome and underrepresented in the core genome. A total of 1190 (59%) of the *N. lactamica *CDSs were present in all the seven completed genomes available at the time of writing (Table 1) and these can be regarded as an estimate of the core genome for these three species (Additional file 7). This may actually be an underestimate of the core genome as this dataset was automatically produced and not manually curated. Therefore mis-annotations in any of the genomes would mean that the genes involved might automatically be excluded. The core genome of *N. meningitidis *alone has been estimated to consist of 1706 genes when the genomes of Z2491, MC58 and FAM18 were examined [8] and approximately 1300 genes when seven meningococcal genomes were examined [65]. The latter estimate is very similar to the core genome proposed for the three species here and, given that the number of core genes in purely meningococcal comparisons is likely to reduce as more genomes become available, suggests that the core genome, amounting to some 60% of the total genome, is common to all three species.

The draft genomes sequences of eight human *Neisseria *commensal species have been compared to eleven previously sequenced *Neisseria *genomes [66] and a *Neisseria *core genome of 896 genes was defined, consisting mainly of housekeeping genes. This analysis, however included *Neisseria elongata, which is *more distantly related to the meningococcus than the other *Neisseria *commensals analysed, and is an unusual member of the genus in that it is rod-shaped, in contrast to other *Neisseria *spp. which are diplococcic. This species also shares fewer core genes with the pathogenic species than the other commensals. Further studies, involving more commensal genomes will help define further the core *Neisseria *genome and may also alter the species definitions of organisms currently defined as *Neisseria*.

For the core genome defined here CDSs ranged from 36.4% to 100% amino acid identity. However, 1180 of these CDSs had greater than 70% amino acid identity among all the genomes examined. The ten CDSs that had less than 70% amino acid identity to the corresponding CDSs in isolate Z2491 included seven that were classified as transport/membrane proteins; one unknown protein; a 3-oxoacyl-(acyl carrier protein) synthase II protein; and putative ribonuclease BN. However, the lower identity was not consistent across the genomes. For example, the transferrin-binding protein B (TbpB) in Z2491 was greater than 70% identical to the corresponding proteins in the other genomes except for the genomes of the two serogroup C meningococci (FAM18 and 053442). Whereas the TbpB in isolate 053442 was 67.3% identical to the Z2491 TbpB, the FAM18 TbpB was only 41.7% identical. Previous work has determined that there are two *tbpB *isotypes, with isotype 1 solely identified among *N. meningitidis *isolates belonging to the ST-11 clonal complex [67], of which isolate FAM18 is a member. The CDS encoding the porin protein PorB also showed differing degrees of identity among the genomes, with the FAM18 porin having the least identity to Z2491 (61.8%). The PorB proteins are known to be diverse [68] with the FAM18 porin belonging to class PorB2 and the Z2491 porin belonging to class PorB3.

**Figure 4:**
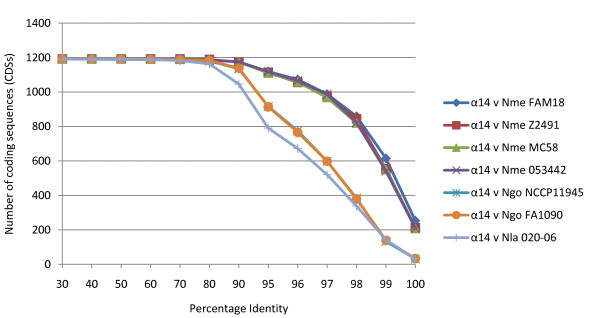
**The percentage identity of CDSs shared by *N. meningitidis *strain α14 and seven completely sequenced *Neisseria *genomes**. Putative orthologues were identified by reciprocal-best-match FASTA searches between α14 and the amino acid sequences of the other genomes using cut-offs of 80% sequence length and 30% identity.

Pair-wise comparisons of the 1190 core CDS sequences from meningococcal isolate α14 against each of the remaining genomes generated a profile of relatedness that was concordant with the microbiological species groups (Figure 4). All five meningococcal isolates were identical at 72 loci, with many of these loci encoding ribosomal proteins (Additional file 7). By contrast, a total of 30 *N. lactamica *020-06 and 29 gonococcal FA1090 CDSs were identical with their orthologues in Z2491. As the threshold of identity was lowered, these relationships were retained, with all four meningococcal genomes showing close relationships to the genome of isolate Z2491 and the *N. lactamica *and gonococcal genomes showing lower numbers of CDSs at each level of sequence identity, with all genomes converging at >70% identity for 1180 CDSs. These relationships were consistent with the CLONALFRAME genealogy obtained from the 19 locus analysis (Figure 1) and provided no support for the idea that the acapsulate ST-53 meningococcal isolate α14 was more closely related to *N. lactamica *and *N. gonorrhoeae *than the encapsulated meningococci [13,65]. Therefore, although 60% of the CDSs present in a given *Neisseria *genome appear to be common to all three species, and there is a high level of shared ancestry among meningococci and *N. lactamica*, the core genomes of each species are characterized by particular sequence poylmorphisms. Again, this is consistent with the results of the 19 locus analysis and provided more evidence for lack of frequent inter-species genetic exchange among the core genomes of these organisms.

### The accessory genome

The remaining 40% of the *N. lactamica *020-06 genome represents the 'accessory genome' made up of elements that are not present in all *Neisseria *isolates. Comparisons with the seven available complete genomes revealed very few genes that were unique to *N. lactamica*, although this list is likely to change as data accumulates from more genomes. These included the *lacZ *and *lacY *genes, which confer the lactose fermenting phenotype after which the bacterium is named, and which replace a haemolysin found in meningococci and gonococci [7]. The low GC content of these genes suggests that they may have been acquired from an unrelated oropharyngeal bacterium. Other genes unique to *N. lactamica*, which may have been horizontally acquired after speciation, include four genes putatively involved in phosphorylcholine biogenesis (*licA*, *licB*, *licC *and *licD*), which may have been acquired by horizontal exchange from *Haemophilus influenzae *[69]; *slpA*, which encodes a putative AAA+ATPase, and *slpB*, which encodes a putative subtilisin-like protease; two low GC content genes that are present in a small number of *N. lactamica *strains and one *Neisseria sicca *strain [11]; and several genes that encode putative adhesins (Table 2).

**Table 2:** Adhesins/haemolysins absent from pathogenic Neisseria

CDS	Length (bp)	% GC content	Note
NLA8290	8964	60.93	CDS immediately upstream encodes a putative haemolysin activator protein. The two genes form a two-partner secretion (TPS) pathway [89].
NLA8550	9141	59.7	No activator protein immediately upstream.
NLA8610	8427	61.49	CDS immediately upstream encodes a putative haemolysin activator protein. The two genes form a two-partner secretion (TPS) pathway [89].
NLA12310	11184	50.17	Adjacent CDS encodes a putative TonB-dependant receptor.
NLA12600	9780	47.05	Has similarity with surface fibril proteins from *H. influenzae *[90].
NLA16480	798	44.98	Immediately downstream is a putative glycosyltransferase unique to *N. lactamica*.

There were several genes not present in meningococci and gonococci which may have been lost by these organisms on speciation, including those encoding: a putative toxin-antitoxin system (RelEB) [70] and seven CDSs encoding hypothetical proteins not found in the pathogenic *Neisseria *upstream of these loci; a putative TonB-dependant receptor that is also present in a number of other *Neisseria *species (*Neisseria cinerea *ATCC14685, *Neisseria sicca *ATCC29256, *Neisseria subflava *NJ9703, *Neisseria flavescens *NRL30031, *Neisseria mucosa *ATCC25996 sequenced at the Genome Sequencing Center at the University of Washington and available at http://www.ncbi.nlm.nih.gov/). The *N. lactamica *genome appears to have two different copies of genes encoding putative L-lactate permeases. One (NLA16970), encodes an L-lactate permease similar to those found in *N. gonorrhoeae *and *N. meningitidis *and the other (NLA5610) is present only in *N. lactamica *020-06 and other genomes of commensal *Neisseria *(*N. lactamica *ATCC 23970, *N. cinerea *ATCC14685, *N. sicca *ATCC29256 *, N. mucosa *ATCC25996, sequenced at The Genome Sequencing Center, University of Washington). Downstream of NLA5610 are genes that encode two putative TonB dependant receptors: NLA5590 which is present in pathogenic and commensal *Neisseria*, and NLA5600, which has only been found in *N. lactamica *020-06 and commensals sequenced at The Genome Sequencing Center, University of Washington *(N. lactamica N. cinerea, N. sicca, N. mucosa, N. subflava, and N. flavescens)*. These two genes may have been deleted from the meningococcal and gonococcal genomes, as the GC content of these sequences in *N. lactamica *020-06 did not suggest horizontal genetic exchange.

Although *N. lactamica *is distinct from *N. gonorrhoeae*, there are some similarities between the two species. The most obvious is the lack of capsule in both *N. lactamica *and *N. gonorrhoeae*. In addition, the putative toxin-antitoxin system FitAB is present in *N. lactamica *and *N. gonorrhoeae *but not *N. meningitidis*. A gonococcal mutant that lacks *fitAB *grows normally extracellularly, but has an accelerated rate of intracellular replication with a concomitant increase in the rate at which this mutant traverses a monolayer of polarized epithelial cells [71]. As *N. meningitidis *lacks both FitA and FitB, intracellular replication and intracellular trafficking may be greater in meningococci. NLA13470 and NLA13480 (putatively *azlC *and *azlD *respectively) are also present in *N. gonorrhoeae*. In *N. meningitidis*, *azlC *is truncated and *azlD *is not present, suggesting this region has been disrupted in *N. meningitidis*. A list of genes present in meningococcal genomes Z2491, MC58, FAM18, and the gonococcal genome FA1090, but absent from *N. lactamica *020-06 is included in Additional file 8.

Out of a total of 134 candidate meningococcal virulence genes proposed previously, 115 (86%) have been shown to be present in the genomes of three meningococcal isolates from asymptomatic carriage (α14, capsule null; α153, serogroup 29E; and α275, serogroup W-135) [13]. A total of 78 (58%) of these genes were present in the *N. lactamica *020-06 genome (Additional file 9). The remaining 56 '*N. meningitidis *specific' genes were not found in all meningococcal genomes and some were present as putative pseudogenes [13]. Functional genes found in all the meningococcal genomes analysed to date but not found in *N. lactamica *020-06 included: *lgtA*, *opcB*, *porA*, *fetB2*, *frpA*, *frpC*, *iga1*, *sodC*, gna1870, *natC *and *nlp*; however, gna1870 appeared to be present in other *N. lactamica *isolates (our unpublished data) and many of the other genes may yet be found in *N. lactamica *isolates that have not been sequenced. There is an *iga2 *gene present in *N. lactamica *(NLA19200); however, this gene has a ~708 bp insertion relative to the *iga2 *gene in the pathogenic *Neisseria *and bears no resemblance to the gene that encodes the IgA protease, which is implicated in pathogenesis.

These analyses confirm that the majority of the accessory genome is shared among all three *Neisseria *species, probably by horizontal genetic exchange, and that a 'pathogenome' of accessory genes, which explains differences in the pathogenic phenotypes among these organisms cannot readily be defined. Some of the shared CDSs have previously been described as 'putative virulence genes' but these are better thought of as genes that confer fitness benefits to transmission only in certain circumstances or genetic backgrounds and which affect the probability of causing invasive disease only incidentally [13]; indeed, many of these genes have now been identified in the complete genome of *N. lactamica *020-06 and other *N. lactamica *isolates [7]. As meningococcal-specific sequences are thought to represent only about 5% of the genome of *N. meningitidis *[72], differences in virulence among isolates and species is likely to be due to subtle changes in their genetic organisation and in their alleles, making the determination of what constitutes a pathogenic phenotype complex for these organisms. This observation is consistent with the conclusions of a previous micro-array-based study, which suggested that a complex integrated network of genes, regulation and diversity of function in common genes could be responsible for the behavioural differences among *N. lactamica*, *N. meningitidis *and *N. gonorrhoeae*. Consequently, for these organisms it is not possible to attribute the emergence of an invasive phenotype to a single event, a single gene set, or loss or gain of a single function [7].

## Conclusion

Comparisons of the genome of *N. lactamica *020-06 with those of the meningococcus and gonococcus provide further detail as to how three closely related biological species, each with distinct genetic and phenotypic characteristics have emerged from a single ancestral population [28]. All three have retained very similar genomes in terms of overall composition, size, and architecture, suggesting that this divergence is relatively recent. The most likely scenario for the emergence of the gonococcus remains the invasion of a single clone into a novel niche, the urogenital tract. This argument is strengthened by the presence of *porA*, gamma-glutamyltranspeptidase (*ggt*) and *opc *pseudogenes in *N. gonorrhoeae *genomes [73-75]. Speciation of *N. lactamica *and *N. meningitidis *has followed a different path, with the two populations diverging without a bottleneck event, therefore retaining substantial shared ancestral diversity. It has been proposed that the species *N. meningitidis *emerged at the same time that meningococcal disease was first described in 1806 [13,76], but this is highly conjectural as commensal meningococci are likely to have been in existence for many thousands of years. It is unclear how gene flow between these groups was disrupted sufficiently for the emergence of distinct species [77], but perhaps this was a consequence of specialisation in the colonisation of different host age groups. There is little evidence of frequent genetic exchange among the present day populations except for genes under positive selection, mostly those encoding variable antigens [54,56]. Occasionally housekeeping alleles adjacent to genes under positive selection are exchanged as a consequence of hitchhiking [54], but overall the species groups are coherent. The observed infrequent inter-species genetic exchange among housekeeping genes is supported by an estimate of the average size of an imported genetic fragment which is 1100 bp [78], larger than the average gene length in the meningococcal genome (852 bp) [35]. If inter-species genetic exchange was frequent among housekeeping genes, gene replacements and therefore the sharing of alleles among species would be common. This clearly is not the case here and proving that horizontal exchange has occurred can be difficult as it requires more evidence than shared polymorphisms alone, which may have been inherited vertically from the ancestral population.

Recombination has erased any phylogenetic signal within each of these species groups at most housekeeping loci. For the single clone origin gonococcus, relatively little variation has accumulated in housekeeping genes but that which has, has been extensively reassorted. In meningococci and *N. lactamica*, extensive genetic diversity, much of it inherited from the common ancestral gene pool, is organised into clonal complexes, each associated with particular alleles at each of the housekeeping gene loci analysed to date. Unlike the polymorphisms, these alleles appear to post-date speciation. The core genome is very similar in terms of orthologous genes among all three species and estimates of the three species and meningococcal core genome of ~1200 and ~1300 [65] CDSs respectively, are also very similar. This suggests that there are few differences in the core genomes of these three species and few core genes are limited to just one species group. This is also true of the accessory genome, the majority of which is shared among the three species. Genetic variation is not, however, evenly distributed among all three species, with the core genomes exhibiting distinct polymorphisms (Figure 4). Thus it appears that sequence variation in housekeeping genes, notwithstanding appreciable shared history, is particular to each species. This may be due to coherence imposed by recombination, or it may represent co-evolution within the housekeeping genes of each species group, which could help to maintain distinct groups if hybrids that contain sequence diversity of more than one group are less fit for host-to-host transmission.

There has been a consistent temptation to map an evolutionary path from the acapsulate *Neisseria *species, including *N. lactamica *and the gonococcus, via capsule null (*cnl*) meningococci to capsulate and therefore invasive meningococci [13]; however, there is no genealogical support for this view (Figure 1) and phylogenetic trees presented as providing evidence have been misinterpreted in that the position of a *cnl *meningococcus marginally closer to a long branch leading to *N. lactamica *and gonococci does not provide strong evidence for this process [13]. It is also the case that members of characteristically *cnl *meningococcal clonal complexes [79], such as the ST-53 complex which is represented in this analysis by the complete genome of isolate α14, can acquire a capsule and cause invasive disease. Indeed, the first representative of the ST-53 complex to be isolated was a serogroup C-expressing meningococcus from a case of invasive disease [80]. The data are, therefore, more consistent with the capsule region genes spreading though the meningococcal population post speciation by an infectious genetic exchange process, which has not reached fixation [81,82]. Thus the *cnl *is the ancestral state of the meningococcal population but meningococci with the *cnl *do not represent ancestors of present day capsulate meningococci.

The search for a 'pathogenome' for the pathogenic *Neisseria *remains frustratingly, perhaps permanently, incomplete. Unlike the enteric bacteria, where transferable genetic elements that encode virulence factors ('pathogenicity associated islands', PAIs), are associated with particular strains that cause given disease syndromes [83], the genetics of pathogenicity in the *Neisseria *is more complex, with pathogenicity-associated genes distributed throughout the genome. Meningococcal population diversity is structured into clonal complexes, some of which, the hyperinvasive lineages, are more likely to cause invasive disease than others [22]. The capsule is the primary virulence determinant, but whilst this is necessary it is not sufficient to cause disease [84]. Other virulence determinants, such as the MDA phage [32] and haemoglobin receptor (HmbR) [85] are widely and unpredictably distributed among meningococci and non-pathogenic *Neisseria*, indicating the hyperinvasive phenotype is probably encoded by different genetic elements in different meningococcal lineages. If, as has been recently suggested, clonal complex structure in the meningococcus, and by extension in *N. lactamica*, is a consequence of the selection of genetic types that are suited to particular micro-niches [86], then the increased probability of invasion will be generated in different lineages by different combinations of genetic traits. If this is indeed the case, then the genetic basis of the meningococcal hyperinvasive phenotype will only become evident by the exploitation of parallel sequencing technologies [87] in whole-genome analyses of large collections of *Neisseria *isolates that are representative of diverse genotypes and phenotypes [88].

## Authors' contributions

MAQ constructed the 020-06 genomic DNA shotgun libraries for sequencing. IC and BW performed the genome assembly and finishing of the genome sequence. JP conceived and oversaw the genome sequencing strategy and oversaw the process. SDB contributed to the shotgun sequencing, genome assembly and annotation procedures. JSB annotated the *N. lactamica *genome and analysed the data. MCJM and JSB wrote the manuscript. GSV annotated the *N. lactamica *genome in terms of repeat families and carried out the comparative analysis of the core gene dataset.

All authors read and approved the final manuscript.

## Supplementary Material

Additional file 1 **Isolates**. Click here for file

Additional file 2 **Genealogical relationships of *Neisseria *isolates inferred from 19 loci using split decomposition and neighbour joining**. A: Neighbour joining. B: Split decomposition. Phylogenies were drawn from concatenated sequences from the 19 housekeeping gene fragments used for the CLONALFRAME tree. Click here for file

Additional file 3 **Neighbour joining trees of the nucleotide sequences from the individual loci: *abcZ, adk, aroE, aspA, carB, dhps***. Nla = red, Nme = blue, Ngo = green. Click here for file

Additional files 4 **Neighbour joining trees of the nucleotide sequences from the individual loci: *fumC, gdh, glnA, gpm, pdhC, pgm***. Nla = red, Nme = blue, Ngo = green Click here for file

Additional files 5 **Neighbour joining trees of the nucleotide sequences from the individual loci: *pilA, pip, ppk, pykA, rpiA, serC, talA***. Nla = red, Nme = blue, Ngo = green. Alleles shared between Nme and Nla = yellow. Click here for file

Additional file 6 **GC deviation in *Neisseria***. Click here for file

Additional file 7 **The 1190 genes common to *N. meningitidis*, *N. gonorrhoeae *and *N. lactamica***. Click here for file

Additional file 8 **CDSs absent from *N. lactamica *020-06**. Click here for file

Additional file 9 **Candidate virulence genes in *N. lactamica***. Click here for file
