# Supplementary figures and images for: Independent evolution of the core and accessory gene sets in the genus Neisseria: insights gained from the genome of Neisseria lactamica isolate 020-06
Source: BMC Genomics. 2010 Nov 23;11:652. doi: 10.1186/1471-2164-11-652 (PMC3091772; doi:10.1186/1471-2164-11-652)

A

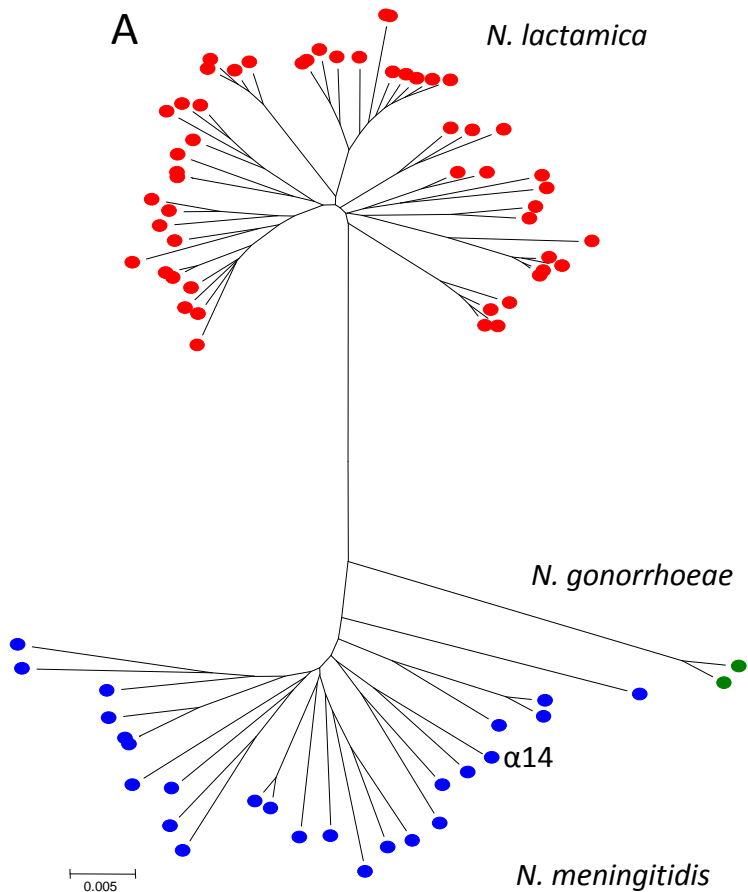

B

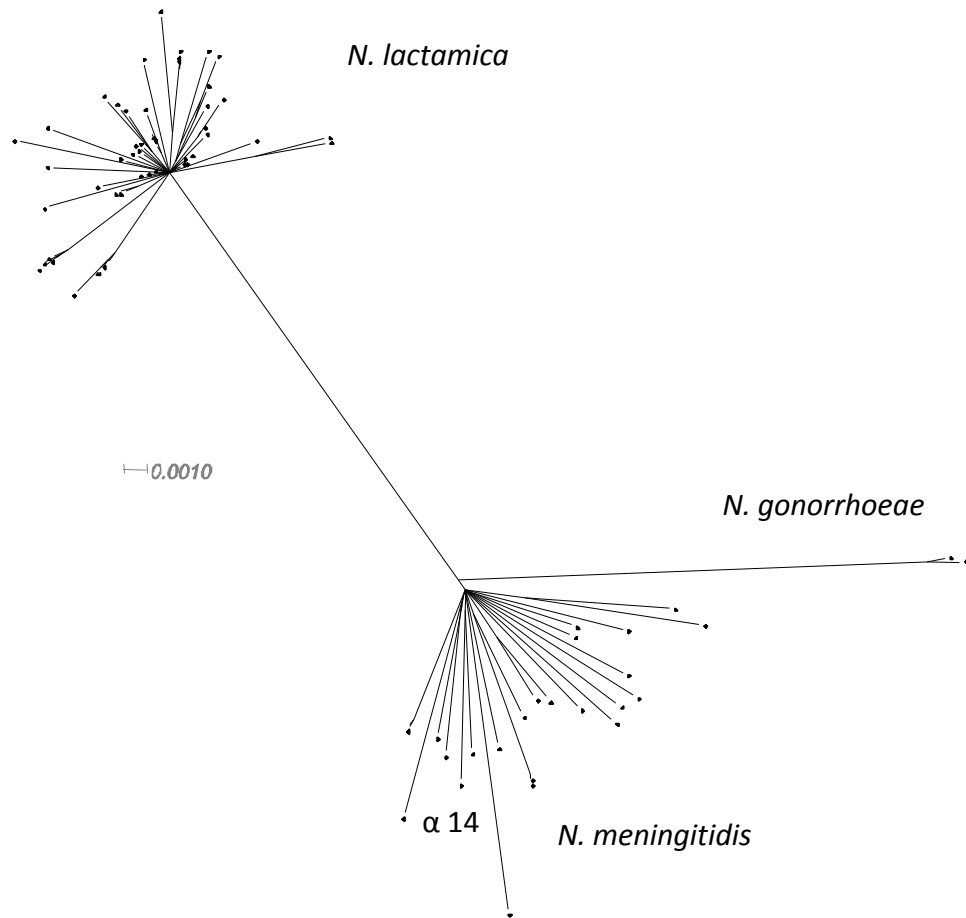

Supplement: Additional file 2 — Genealogical relationships of Neisseria isolates inferred from 19 loci using split decomposition and neighbour joining. A: Neighbour joining. B: Split decomposition. Phylogenies were drawn from concatenated sequences from the 19 housekeeping gene fragments used for the CLONALFRAME tree. [file 1471-2164-11-652-S2.PDF]

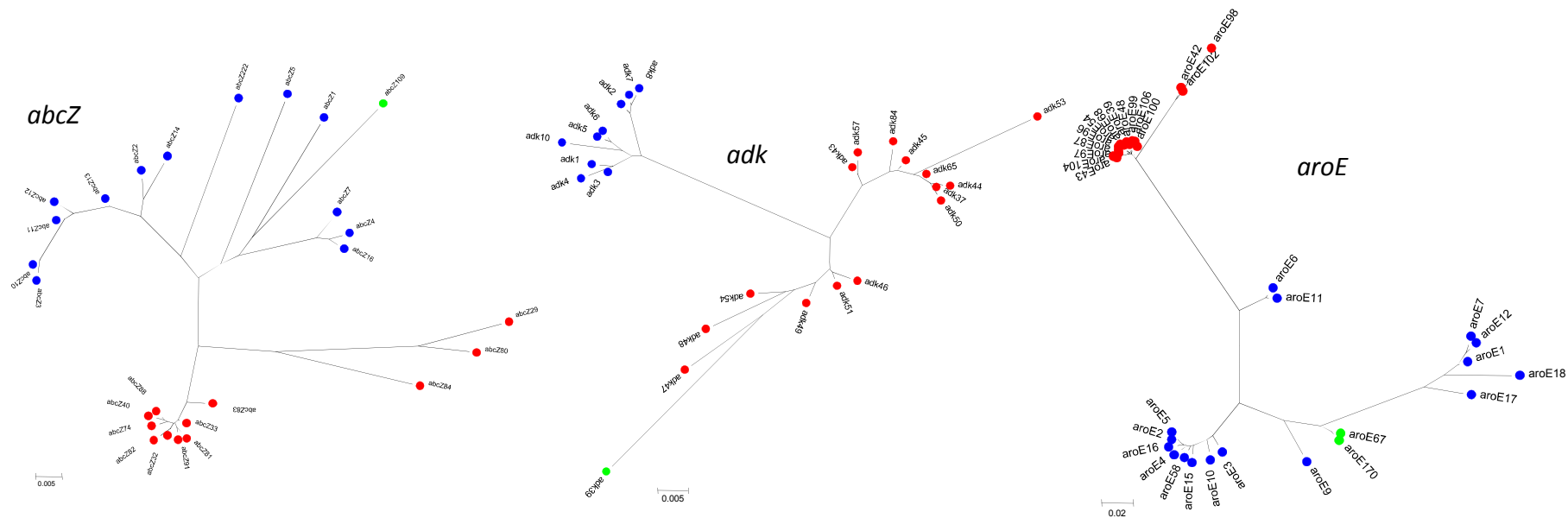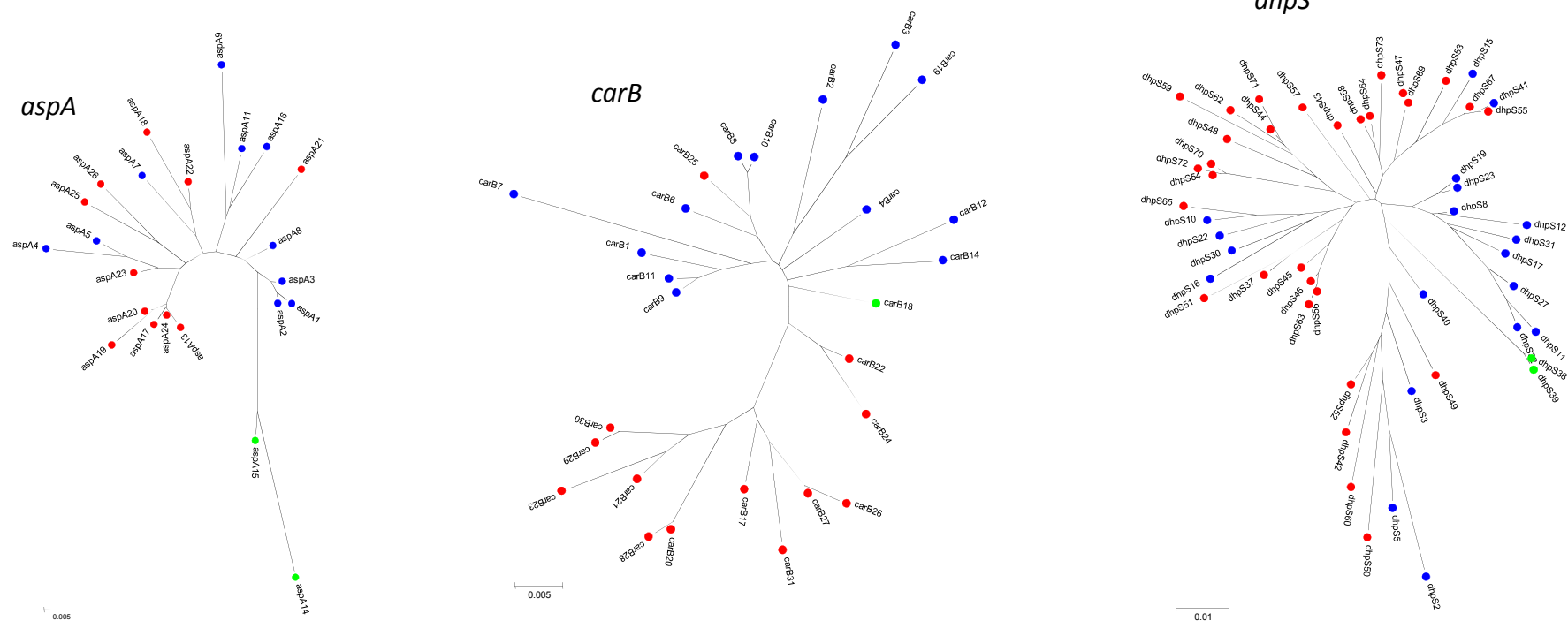

Supplement: Additional file 3 — Neighbour joining trees of the nucleotide sequences from the individual loci: abcZ, adk, aroE, aspA, carB, dhps. Nla = red, Nme = blue, Ngo = green. [file 1471-2164-11-652-S3.PDF]

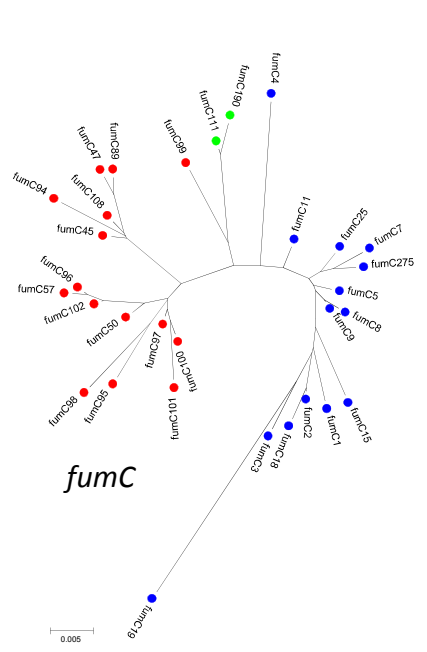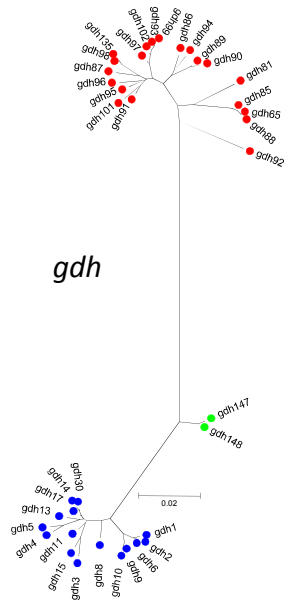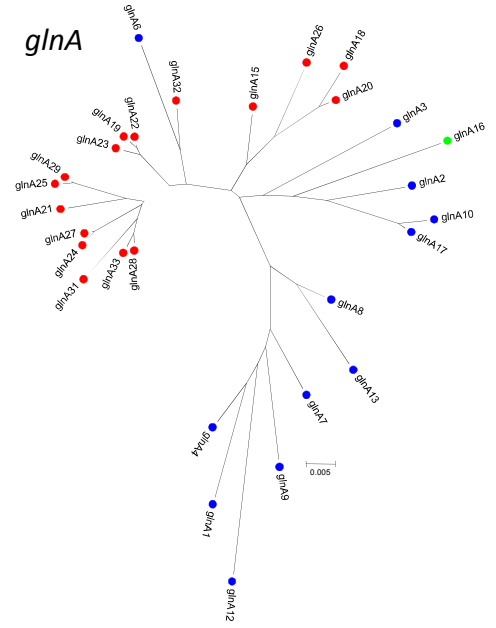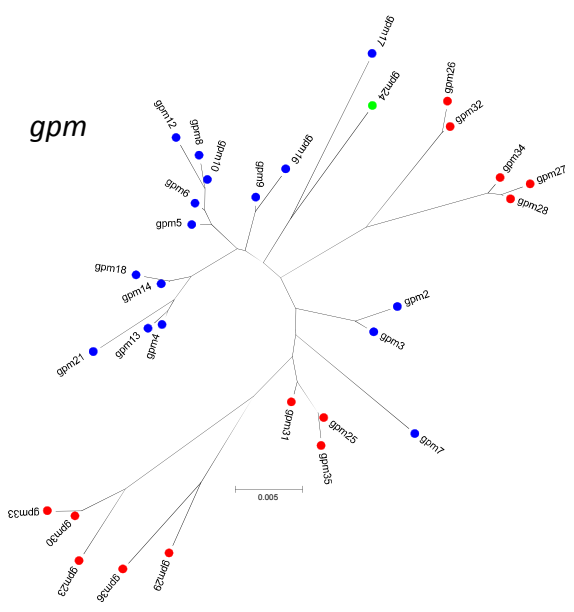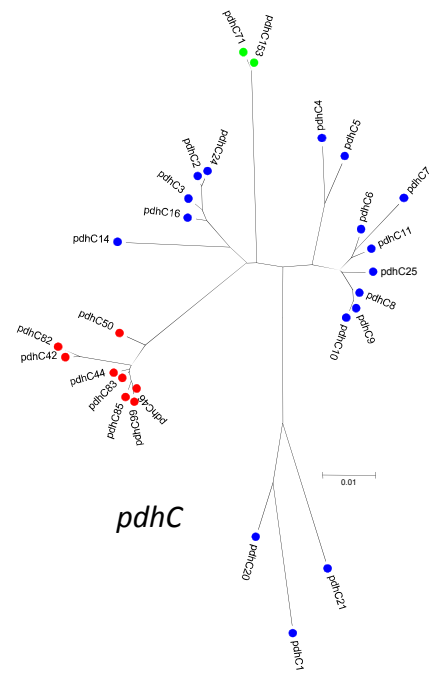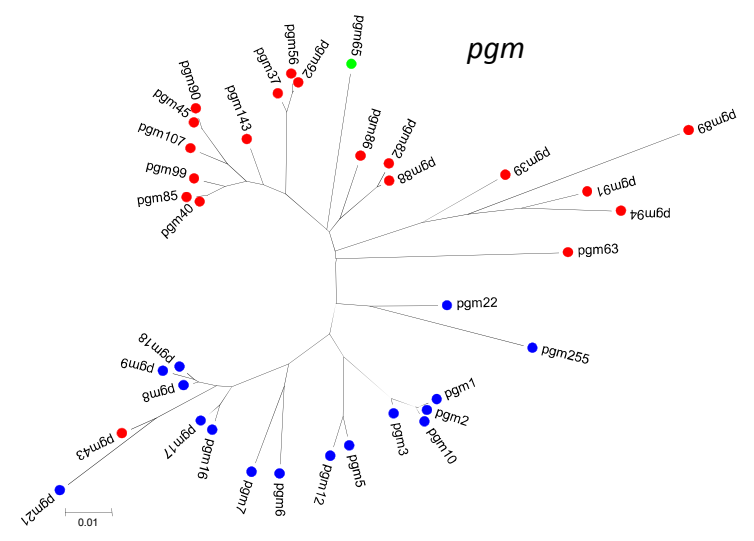

Supplement: Additional files 4 — Neighbour joining trees of the nucleotide sequences from the individual loci: fumC, gdh, glnA, gpm, pdhC, pgm. Nla = red, Nme = blue, Ngo = green [file 1471-2164-11-652-S4.PDF]

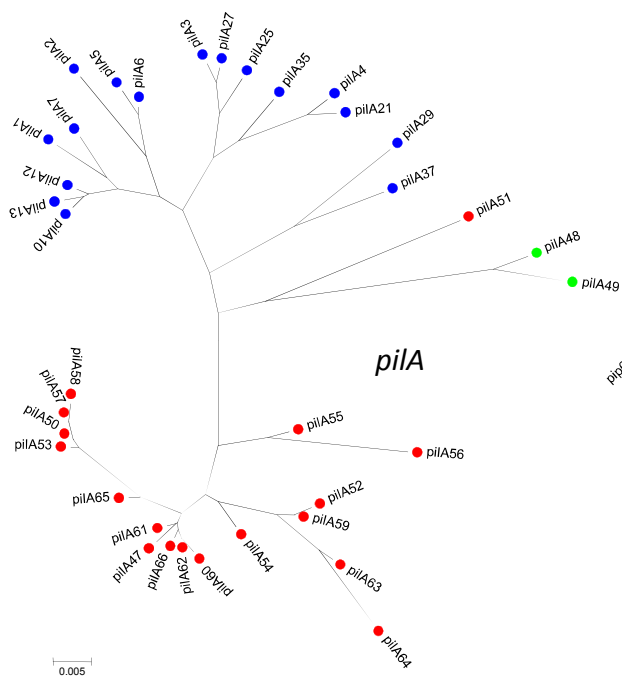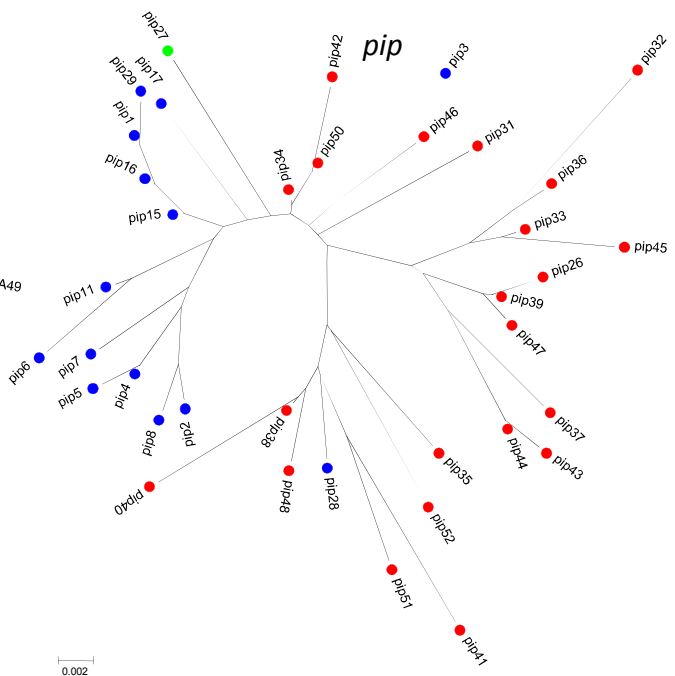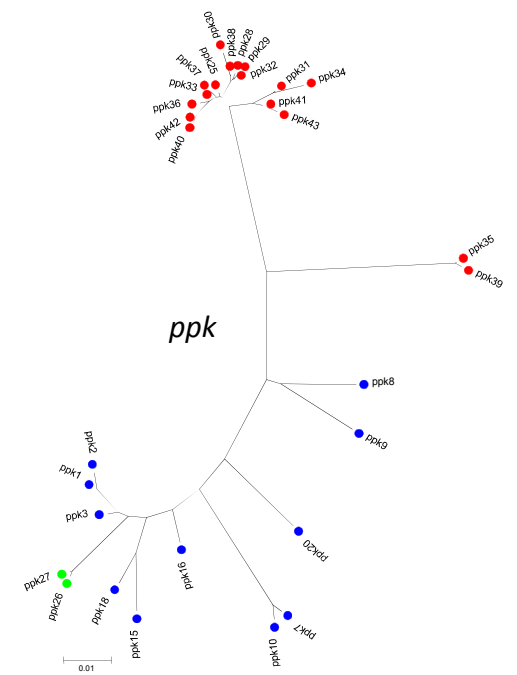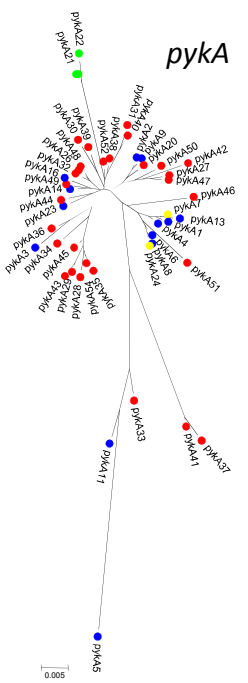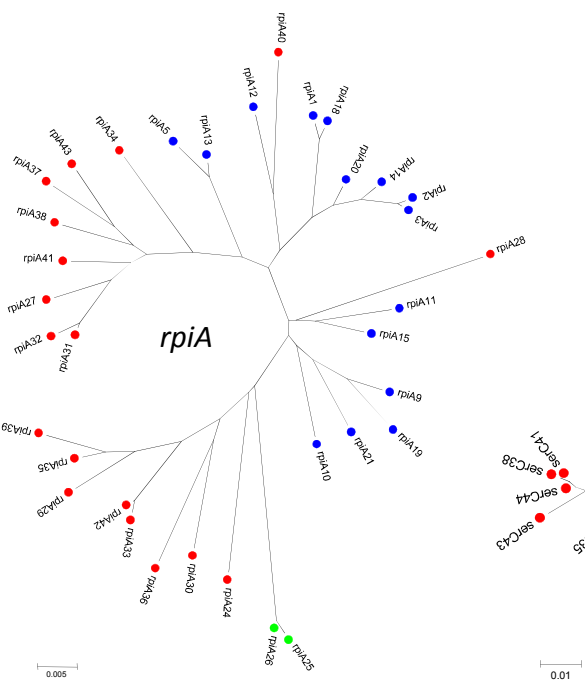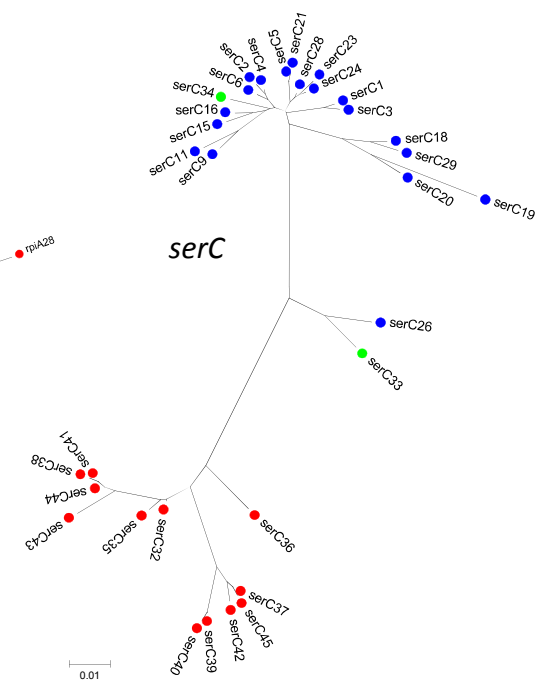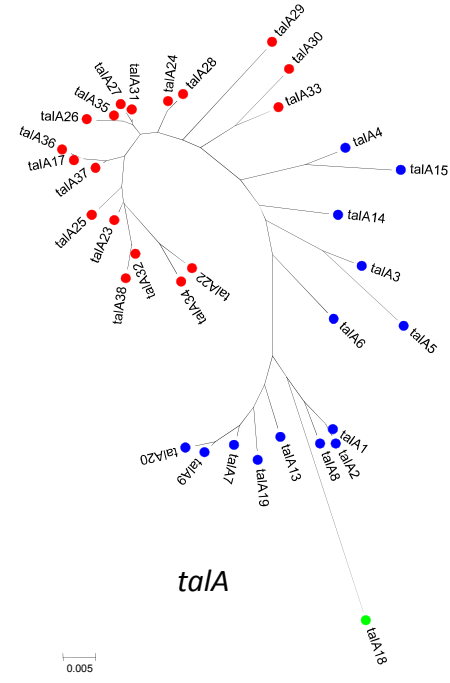

Supplement: Additional files 5 — Neighbour joining trees of the nucleotide sequences from the individual loci: pilA, pip, ppk, pykA, rpiA, serC, talA. Nla = red, Nme = blue, Ngo = green. Alleles shared between Nme and Nla = yellow. [file 1471-2164-11-652-S5.PDF]

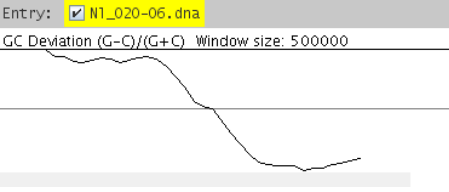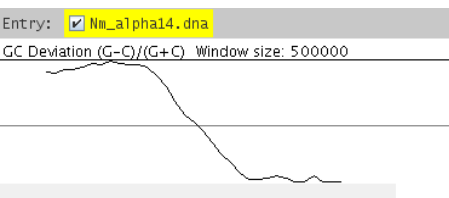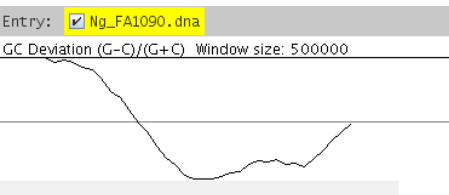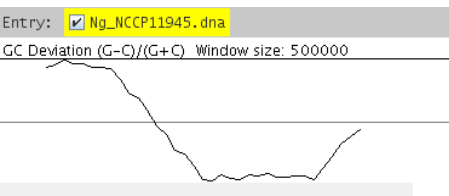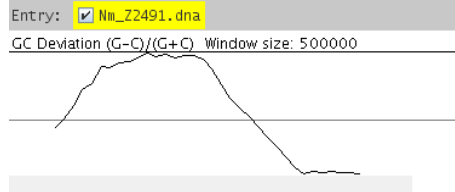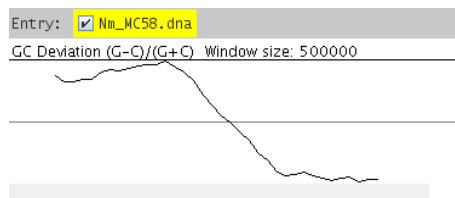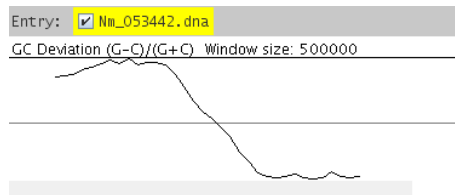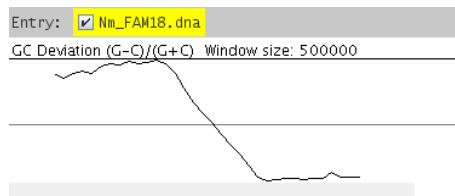

Supplement: Additional file 6 — GC deviation in Neisseria. [file 1471-2164-11-652-S6.PDF]
