# Supplementary material for: Independent evolution of the core and accessory gene sets in the genus Neisseria: insights gained from the genome of Neisseria lactamica isolate 020-06
Source: BMC Genomics. 2010 Nov 23;11:652. doi: 10.1186/1471-2164-11-652 (PMC3091772; doi:10.1186/1471-2164-11-652)
Supplement: Additional file 9 — Candidate virulence genes in N. lactamica. [file 1471-2164-11-652-S9.DOC]

****Table 3. Distribution of genes described as candidate virulence genes in sequenced genomes of** N. meningitidis and N. lactamica 020-06**

| Synonym | Function | COG | | | α14 | | | | | Z2491 | | | | MC58 | | | | | FAM18 | | | | α153^†^ | | | | α275^†^ | | | | Nla 020-06 |
| --- | --- | --- | --- | --- | --- | --- | --- | --- | --- | --- | --- | --- | --- | --- | --- | --- | --- | --- | --- | --- | --- | --- | --- | --- | --- | --- | --- | --- | --- | --- | --- |
| LOS synthesis | | | | | | | | | | | | | | | | | | | | | | | | | | | | | | | |
| envA | UDP-3-O-acyl-N-acetylglucosamine deacetylase | M | | | | NMO1987 | | | | NMA0263 | | | | NMB0017 | | | | NMC0001 | | | | | NME_1824 | | | | NMW_0042 | | | | NLA0010 |
| kdtA | 3-Deoxy-d-manno-octulosonic-acid transferase | M | | | | NMO0002 | | | | NMA0261 | | | | NMB0014 | | | | NMC2152 | | | | | NME_1827 | | | | - | | | | NLA20660 |
| lgtA | Lacto-N-neotetraose biosynthesis glycosyl transferase | M | | | | NMO0241 | | | | NMA0524 | | | | NMB1929 | | | | NMC1902 | | | | | NME_1448 | | | | NMW_0807 | | | | - |
| lgtB | Lacto-N-neotetraose biosynthesis glycosyl tranferase | M | | | | NMO0242 | | | | NMA0525 | | | | NMB1928 | | | | NMC1901 | | | | | NME_1449 | | | | NMW_0806 | | | | NLA18710 |
| lgtE | Lacto-N-neotetraose biosynthesis glycosyl transferase | M | | | | - | | | | - | | | | NMB1926 | | | | - | | | | | - | | | | - | | | | - |
| lgtF | β1;4 glucosyltransferase | M | | | | NMO1519 | | | | NMA1958 | | | | NMB1704 | | | | NMC1618 | | | | | NME_2238 | | | | NMW_0056 | | | | NLA15680 |
| lpxA | Acyl-UDP-N-acetylglucosamine O-acyltransferase | M | | | | NMO1860 | | | | NMA0090 | | | | NMB0178 | | | | NMC0168 | | | | | - | | | | NMW_0980 | | | | NLA1250 |
| lpxB | Lipid A disaccharide synthase | M | | | | NMO1840 | | | | NMA0069 | | | | NMB0199 | | | | NMC0191 | | | | | NME_0996 | | | | NMW_0381 | | | | NLA1580 |
| lpxD | UDP-3-O-glucosamine N-acyltransferase | M | | | | NMO1858 | | | | NMA0087 | | | | NMB0180 | | | | NMC0171 | | | | | NME_0965 | | | | NMW_0978 | | | | NLA1280 |
| lst | α-2;3-sialyltransferase | - | | | | NMO0817 | | | | NMA1118 | | | | NMB0922 | | | | NMC0899 | | | | | NME_0163 | | | | NMW_1776 | | | | NLA13410 |
| pgm | Phosphomannomutase | G | | | | NMO0678 | | | | NMA1001 | | | | NMB0790 | | | | NMC0743 | | | | | NME_1183 | | | | NMW_0149 | | | | NLA14390 |
| rfaC | Lipopolysaccharide heptosyltransferase I | M | | | | NMO0022 | | | | NMA0243 | | | | NMB2156 | | | | NMC2134 | | | | | NME_1849 | | | | NMW_0841 | | | | NLA20480 |
| rfaD | ADP-l-glycero-d-mannoheptose-6-epimerase | M, G | | | | NMO0712 | | | | NMA1037 | | | | NMB0828 | | | | NMC0773 | | | | | NME_1223 | | | | NMW_0857 | | | | NLA14080 |
| rfaE | ADP-heptose synthase | M | | | | NMO0711 | | | | NMA1034 | | | | NMB0825 | | | | NMC0769 | | | | | NME_1219 | | | | NMW_0854 | | | | NLA14110 |
| rfaF | ADP-heptose--LPS heptosyltransferase II | M | | | | NMO1348 | | | | NMA1727 | | | | NMB1527 | | | | NMC1456 | | | | | NME_1271 | | | | NMW_0556 | | | | NLA7500 |
| rfaK | α1;2 N-acetylglucosamine transferase | M | | | | NMO1520 | | | | NMA1959 | | | | NMB1705 | | | | NMC1619 | | | | | NME_2239 | | | | NMW_0055 | | | | NLA5670 |
| Capsule expression | | | | | | | | | | | | | | | | | | | | | | | | | | | | | | | |
| ctrA | Capsule polysaccharide export outer membrane protein | | | M | | | | - | | | | | NMA0198 | | NMB0071 | | | | | NMC0055 | | | | NME_2072 | | | | NMW_2125 | | | - |
| ctrB | Capsule polysaccharide export inner-membrane protein | | | M | | | | - | | | | | NMA0197 | | NMB0072 | | | | | NMC0056 | | | | NME_2073 | | | | NMW_2124 | | | - |
| ctrC | Capsule polysaccharide export inner-membrane protein | | | G, M | | | | - | | | | | NMA0196 | | NMB0073 | | | | | NMC0057 | | | | NME_2074 | | | | NMW_2123 | | | - |
| ctrD | Capsule polysaccharide export ATP-binding protein | | | G, M | | | | - | | | | | NMA0195 | | NMB0074 | | | | | NMC0058 | | | | NME_2075 | | | | NMW_2122 | | | - |
| lipA | Capsule polysaccharide modification protein | | | I | | | | - | | | | | NMA0186 | | NMB0082 | | | | | NMC0066 | | | | NME_2084 | | | | NMW_2113 | | | - |
| lipB | Capsule polysaccharide modification protein | | | I | | | | - | | | | | NMA0185 | | NMB0083 | | | | | NMC0067 | | | | NME_2085 | | | | NMW_2112 | | | - |
| sacA | UDP-N-acetyl-d-glucosamine 2-epimerase | | | M | | | | - | | | | | NMA0199 | | - | | | | | - | | | | - | | | | - | | | - |
| sacB | Capsule biosynthesis protein | | | - | | | | - | | | | | NMA0200 | | - | | | | | - | | | | - | | | | - | | | - |
| sacC | Capsule biosynthesis protein | | | - | | | | - | | | | | NMA0201 | | - | | | | | - | | | | - | | | | - | | | - |
| sacD | Capsule biosynthesis protein | | | M | | | | - | | | | | NMA0202 | | - | | | | | - | | | | - | | | | - | | | - |
| siaA | Polysialic acid capsule biosynthesis protein SynX | | | M | | | | - | | | | | - | | NMB0070 | | | | | NMC0054 | | | | - | | | | NMW_2126 | | | - |
| siaB | Polysialic acid capsule biosynthesis protein SiaB | | | M | | | | - | | | | | - | | NMB0069 | | | | | NMC0053 | | | | - | | | | - | | | - |
| siaC | Polysialic acid capsule biosynthesis protein SiaC | | | M | | | | - | | | | | - | | NMB0068 | | | | | NMC0052 | | | | - | | | | NMW_2127 | | | - |
| siaDb/siaDc | Polysialic acid capsule biosynthesis protein SiaD | | | - | | | | - | | | | | - | | NMB0067 | | | | | NMC0051 | | | | - | | | | NMW_2128 | | | - |
| Adhesins | | | | | | | | | | | | | | | | | | | | | | | | | | | | | | | |
| nadA | Quinolinate synthetase A | | H | | | | NMO1648 | | | | NMA2090 | | | | | NMB0394 | | | | | NMC1772 | | | | NME_0804 | | | | NMW_0730 | | NLA4460 |
| omp85 | Probable surface antigen | | M | | | | NMO1856 | | | | NMA0085 | | | | | NMB0182 | | | | | NMC0173 | | | | NME_0967 | | | | NMW_0976 | | NLA1300 |
| ompH | Putative outer membrane protein | | M | | | | NMO1857 | | | | NMA0086 | | | | | NMB0181 | | | | | NMC0172 | | | | NME_0966 | | | | NMW_0977 | | NLA1290 |
| opa | Opacity protein and related surface antigens | | M | | | | NMO0821* ^‡^ | | | | - | | | | | NMB0926* | | | | | NMC0903 | | | | NME_1925 | | | | NMW_0456 | | - |
| opa | Opacity protein and related surface antigens | | M | | | | - | | | | NMA1676* | | | | | NMB1465* | | | | | NMC1403 | | | | NME_0431 | | | | NMW_0347 | | NLA10410 |
| opa | Opacity protein and related surface antigens | | M | | | | NMO1454* | | | | NMA1890* | | | | | NMB1636* | | | | | NMC1551 | | | | NME_0192 | | | | NMW_0426 | | - |
| opa | Opacity protein and related surface antigens | | M | | | | NMO1598* | | | | NMA2042 | | | | | NMB0442* | | | | | NMC1719 | | | | NME_1625 | | | | NMW_0958 | | - |
| opcA | Outer membrane protein precursor | | - | | | | - | | | | NMA1251 | | | | | NMB1053 | | | | | - | | | | NME_1303 | | | | NMW_1406 | | - |
| opcB | Outer membrane protein precursor | | - | | | | NMO1736* | | | | NMA2183* | | | | | NMB0304* | | | | | NMC1877* | | | | NME_2335 | | | | NMW_0644 | | - |
| pglA | Pilin glycosylation protein | | M | | | | NMO1820 | | | | NMA0048 | | | | | NMB0218 | | | | | NMC0213* | | | | NME_0675 | | | | NMW_1718 | | NLA1820* |
| pglB | Pilin glycosylation protein | | M | | | | NMO0357 | | | | NMA0639 | | | | | NMB1820 | | | | | - | | | | NME_2153 | | | | - | | NLA17580 |
| pglC | Pilin glycosylation protein | | M | | | | NMO0356 | | | | NMA0638 | | | | | NMB1821 | | | | | NMC0397 | | | | NME_2154 | | | | NMW_1785 | | NLA17590 |
| pglD | Pilin glycosylation protein | | M, G | | | | NMO0355 | | | | NMA0637 | | | | | NMB1822 | | | | | NMC0396 | | | | NME_2155 | | | | NMW_1784 | | NLA1600 |
| pilC1 | Pilus-associated protein | | N | | | | - | | | | NMA0609 | | | | | NMB1847* | | | | | NMC0371 | | | | NME_2193 | | | | NMW_0483 | | - |
| pilC2 | Fimbrial assembly protein | |  | | | | NMO1960 | | | | NMA0293* | | | | | NMB0049* | | | | | NMC0033 | | | | - | | | | NMW_0050 | | - |
| pilE | Fimbrial protein precursor (pilin) | | N, U | | | | NMO1986 | | | | NMA0264 | | | | | NMB0018 | | | | | - | | | | NME_1823 | | | | NMW_0247 | | - |
| pilF | Pilus biogenesis protein | | N, U | | | | NMO1712 | | | | NMA2159 | | | | | NMB0329 | | | | | NMC1844 | | | | NME_0721 | | | | NMW_0528 | | NLA3860 |
| pilG | Type IV pilus assembly protein | | N, U | | | | NMO1708 | | | | NMA2155 | | | | | NMB0333 | | | | | NMC1838 | | | | NME_0726 | | | | NMW_0532 | | NLA3900 |
| pilT-1 | Twitching motility protein | | N, U | | | | NMO1957 | | | | NMA0218 | | | | | NMB0052 | | | | | NMC0036 | | | | NME_2286 | | | | NMW_1290 | | NLA0220 |
| pilT-2 | Ttwitching motility-like protein | |  | | | | NMO0657 | | | | NMA0979 | | | | | NMB0768 | | | | | NMC0721 | | | | NME_0462 | | | | NMW_1079 | | NLA14620 |
| pilV | Type IV pilus assembly protein | | N, U | | | | NMO0764 | | | | NMA1107 | | | | | NMB0887 | | | | | NMC0828 | | | | NME_2102 | | | | NMW_0243 | | NLA13520 |
| porA | Outer membrane porin protein precursor | | M | | | | NMO1262 | | | | NMA1642 | | | | | NMB1429 | | | | | NMC1364 | | | | NME_0491 | | | | NMW_1171 | | - |
| rmpM | Class 4 outer membrane protein | | M | | | | NMO1657 | | | | NMA2105 | | | | | NMB0382 | | | | | NMC1783 | | | | NME_0791 | | | | NMW_0714 | | NLA4380 |
|  | Haemagglutinin/Haemolysin-related protein | | U | | | | - | | | | - | | | | | NMB0493 | | | | | - | | | | - | | | | - | | - |
|  | Haemolysin activator-related protein | | U | | | | NMO0397 | | | | NMA0687 | | | | | NMB0496 | | | | | NMC0443 | | | | NME_0859 | | | | NMW_2277 | | NLA8280 |
|  | Haemagglutinin/Haemolysin-related protein | | U | | | | - | | | | - | | | | | NMB1214 | | | | | - | | | | - | | | | - | | NLA10070 |
|  | Haemagglutinin/Haemolysin-related protein | | U | | | | - | | | | - | | | | | NMB1768 | | | | | - | | | | - | | | | - | | - |
|  | Haemagglutinin/Haemolysin-related protein | | U | | | | NMO0398 | | | | NMA0688 | | | | | NMB0497 | | | | | NMC0444 | | | | NME_0860 | | | | NMW_2276 | | - |
|  | Haemolysin activation protein | | U | | | | NMO0397 | | | | NMA0687 | | | | | NMB1780 | | | | | NMC0443 | | | | NME_0859 | | | | NMW_2277 | | - |
| **Iron uptake systems** | | | | | | | | | | | | | | | | | | | | | | | | | | | | | | | |
| bcp | Bacterioferritin comigratory protein | | O | | | | | | NMO0641 | | | NMA0963 | | | | | NMB0750 | | | | | NMC0704 | | | | NME_0295 | | | | NMW_0027 | NLA14830 |
| bfrA | Bacterioferritin | | P | | | | | | NMO1021 | | | NMA1377 | | | | | NMB1207 | | | | | NMC1108 | | | | NME_2298 | | | | NMW_1230 | NLA10020 |
| bfrB | Bacterioferritin | | P | | | | | | NMO1020 | | | NMA1376 | | | | | NMB1206 | | | | | NMC1107 | | | | NME_2299 | | | | NMW_1231 | NLA10020 |
| fbpA | Iron transport system substrate-binding protein | | P | | | | | | NMO0521 | | | NMA0844 | | | | | NMB0634 | | | | | NMC0578 | | | | NME_1345 | | | | NMW_0513 | NLA15970 |
| fbpB | ABC-type Fe^3+^ transport system; permease component | | P | | | | | | NMO0520 | | | NMA0843 | | | | | NMB0633 | | | | | NMC0577 | | | | NME_1346 | | | | NMW_0512 | NLA15980 |
| fetB2 | Periplasmic binding protein; ABC transporter | | P | | | | | | NMO0287 | | | NMA0577 | | | | | NMB1880 | | | | | NMC0340 | | | | NME_1546 | | | | NMW_0889 | - |
| fur | Ferric uptake regulation protein | | P | | | | | | NMO1835 | | | NMA0064 | | | | | NMB0205 | | | | | NMC0197 | | | | NME_1000 | | | | NMW_0386 | NLA1630 |
| hmbR | Haemoglobin receptor | | P | | | | | | NMO1486 | | | NMA1925* | | | | | NMB1668 | | | | | NMC1586 | | | | NME_2201 | | | | - | - |
|  | Iron ABC transport system | | P | | | | | | NMO0175 | | | NMA0450 | | | | | NMB1991 | | | | | NMC1966 | | | | NME_2255 | | | | NMW_1208 | NLA19340 |
|  | Iron ABC transport system | | P | | | | | | NMO0176 | | | NMA0451 | | | | | NMB1990 | | | | | NMC1965 | | | | NME_2254 | | | | NMW_1207 | NLA19330 |
|  | Iron ABC transport system | | P | | | | | | NMO0178 | | | NMA0452 | | | | | NMB1989 | | | | | NMC1964 | | | | NME_2252 | | | | NMW_1206 | NLA19320 |
| lbpA | Lactoferrin binding protein A | | P | | | | | | NMO0579 | | | NMA1739 | | | | | NMB1540 | | | | | NMC1468 | | | | NME_0340 | | | | NMW_0517 | NLA15490 |
| lbpB | Lactoferrin-binding protein B | | R | | | | | | NMO0578 | | | NMA1740 | | | | | NMB1541 | | | | | NMC1469 | | | | NME_0339 | | | | NMW_0518 | NLA15500 |
| tbp1 | Transferrin-binding protein A | | P | | | | | | NMO1581 | | | NMA2024 | | | | | NMB0461 | | | | | NMC1690 | | | | NME_1606 | | | | NMW_0293 | NLA4990 |
| tbp2 | Transferrin-binding protein B | | - | | | | | | NMO1582 | | | NMA2025 | | | | | NMB0460 | | | | | NMC1691 | | | | NME_1607 | | | | NMW_0295 | NLA4980 |
|  | TonB-dependent receptor | | P | | | | | | - | | | NMA1663* | | | | | NMB1449* | | | | | - | | | | - | | | | NMW_0547 | NLA8780 * |
|  | TonB-dependent receptor | | P | | | | | | NMO1744* | | | NMA2193 | | | | | NMB0293 | | | | | NMC1887 | | | | NME_2111 | | | | NMW_1049 | - |
|  | TonB-dependent receptor | | P | | | | | | NMO0285 | | | NMA0575 | | | | | NMB1882 | | | | | NMC0338 | | | | NME_1544 | | | | NMW_2094 | NLA5590 |
|  | TonB-dependent receptor | | P | | | | | | NMO0332 | | | - | | | | | NMB1829 | | | | | NMC0387 | | | | - | | | | - | NLA17710 |
|  | TonB-dependent receptor | | P | | | | | | NMO1183 | | | NMA1558 | | | | | NMB1346* | | | | | NMC1282 | | | | NME_1132 | | | | NMW_1263 | NLA11790 |
|  | Transferrin binding protein | | - | | | | | | NMO0042 | | | NMA0299 | | | | | NMB2132 | | | | | NMC2109 | | | | NME_1876 | | | | NMW_2154 | NLA20270 |
| tspA | Neisseria-specific antigen protein | | N, U | | | | | | NMO1699 | | | NMA2146 | | | | | NMB0341 | | | | | NMC1829 | | | | NME_0736 | | | | NMW_0542 | NLA4020 |
| tspB | TspB protein | | D | | | | | | - | | | NMA1797 | | | | | NMB1548 | | | | | NMC1866 | | | | - | | | | - | NLA12880 |
| **Putative toxins** | | | | | | | | | | | | | | | | | | | | | | | | | | | | | | | |
| frpA | FrpA/C-related protein | | Q | | | | | | - | | | - | | | | | NMB1409 | | | | | - | | | | - | | | | NMW_0217 | - |
| frpA | RTX family exoprotein | | Q | | | | | | - | | | NMA1625 | | | | | NMB1405 | | | | | - | | | | NME_0326 | | | | NMW_1283 | - |
| frpA | RTX-family exoprotein | | Q | | | | | | NMO0470 | | | NMA0787* | | | | | NMB0585 | | | | | NMC0527 | | | | NME_2069 | | | | - | - |
| frpA | FrpA/C-related protein; truncated | | Q | | | | | | NMO1245 | | | NMA1623 | | | | | NMB1403 | | | | | - | | | | NME_0327 | | | | NMW_1282 | - |
| frpC | FrpC operon protein | | - | | | | | | - | | | - | | | | | NMB1412 | | | | | - | | | | NME_2066 | | | | - | - |
| frpC | FrpC operon protein | | - | | | | | | - | | | - | | | | | NMB1414 | | | | | NMC1344 | | | | NME_2068 | | | | - | - |
| frpC | Hypothetical protein NMA2123 | | Q | | | | | | NMO1677 | | | NMA2123 | | | | | NMB0365 | | | | | NMC1805 | | | | NME_0108 | | | | NMW_1477 | - |
| frpC | FrpC operon protein | | - | | | | | | NMO0469 | | | - | | | | | NMB0584 | | | | | NMC0526 | | | | NME_2068 | | | | NMW_1891 | - |
| frpC | Putative RTX-family exoprotein | | Q | | | | | | NMO1247 | | | NMA1626 | | | | | NMB1415 | | | | | NMC1345 | | | | NME_2069 | | | | - | - |
| frpC | FrpC operon protein | | - | | | | | | NMO1678 | | | NMA2124 | | | | | NMB0364 | | | | | NMC1806 | | | | NME_0107 | | | | NMW_1476 | - |
|  | Putative toxin-activating protein | | O | | | | | | - | | | - | | | | | NMB1210 | | | | | - | | | | - | | | | - | NLA10050 |
|  | Putative toxin-activating protein | | O | | | | | | - | | | - | | | | | NMB1763 | | | | | - | | | | - | | | | - | - |
| **Defensins** | | | | | | | | | | | | | | | | | | | | | | | | | | | | | | | |
| dsbA | Thiol:disulfide interchange protein DsbA | | O, C | | | | | | NMO1633 | | | NMA2078 | | | | | NMB0407 | | | | | NMC1760 | | | | NME_1755 | | | | NMW_1612 | NLA4570 |
| dsbA | Thiol:disulfide interchange protein DsbA | | O, C | | | | | | NMO1743 | | | NMA2191 | | | | | NMB0294 | | | | | NMC1885 | | | | NME_2108 | | | | NMW_1052 | - |
| dsbA | Thiol:disulfide interchange protein DsbA | | O, C | | | | | | NMO1761 | | | NMA2209 | | | | | NMB0278 | | | | | NMC0273 | | | | NME_2128 | | | | NMW_2372 | NLA19860 |
| iga | IgA-specific serine endopeptidase | | M, U | | | | | | NMO0592 | | | NMA0905 | | | | | NMB0700 | | | | | NMC0651 | | | | NME_1078 | | | | NMW_1850 | - |
| kat | Catalase | | P | | | | | | NMO1822 | | | NMA0050 | | | | | NMB0216 | | | | | NMC0211 | | | | NME_0679 | | | | NMW_1715 | NLA1790 |
| sodB | Superoxide dismutase | | P | | | | | | NMO0761 | | | NMA1104 | | | | | NMB0884 | | | | | NMC0825 | | | | NME_2345 | | | | NMW_0240 | NLA13550 |
| sodC | Superoxide dismutase | | P | | | | | | NMO1240 | | | NMA1617 | | | | | NMB1398 | | | | | NMC1339 | | | | NME_0332 | | | | NMW_1276 | - |
| **Others** | | | | | | | | | | | | | | | | | | | | | | | | | | | | | | | |
|  | Twitching motility protein | | N, U | | | | | | NMO1958 | | | NMA0219 | | | | | NMB0051 | | | | | NMC0035 | | | | NME_2287 | | | | NMW_1289 | NLA0210 |
|  | VapD-related protein | | S | | | | | | - | | | - | | | | | NMB1753 | | | | | - | | | | - | | | | - | NLA8850 |
|  | Hypothetical protein NMB0065 | | - | | | | | | - | | | - | | | | | NMB0065 | | | | | NMC0049* | | | | - | | | | - | - |
|  | Putative ClpP class peptidase | | O, U | | | | | | NMO0047 | | | NMA0305 | | | | | NMB2127 | | | | | NMC2103 | | | | NME_1883 | | | | NMW_2147 | NLA20220 |
|  | Putative lipoprotein | | P | | | | | | NMO0225 | | | NMA0506 | | | | | NMB1946 | | | | | NMC1917 | | | | NME_0028 | | | | NMW_2495 | NLA18880 |
|  | Putative periplasmic solute binding protein | | P | | | | | | NMO0471 | | | NMA0789 | | | | | NMB0586 | | | | | NMC0528 | | | | NME_0078 | | | | NMW_0142 | NLA16470 |
|  | Putative autotransporter adhesin | | U, W | | | | | | NMO0900 | | | NMA1200 | | | | | NMB0992 | | | | | NMC0978 | | | | NME_0316 | | | | NMW_2010 | - |
|  | Hly-III related protein | | R | | | | | | NMO1464 | | | NMA1900 | | | | | NMB1646 | | | | | NMC1560 | | | | NME_0175 | | | | NMW_0166 | - |
|  | Putative periplasmic type I secretion system protein | | M | | | | | | NMO1556 | | | NMA1996 | | | | | NMB1738 | | | | | NMC1658 | | | | NME_1148 | | | | NMW_0992 | - |
| dca/pptA | Putative membrane-associated sulfatase | | R | | | | | | NMO1625 | | | NMA2069* | | | | | NMB0415* | | | | | NMC1750 | | | | NMW_1746 | | | | NMW_1604 | - |
| fabZ | 3R-hydroxymyristoyl ACP dehydrase | | I | | | | | | NMO1859 | | | NMA0088 | | | | | NMB0179 | | | | | NMC0170 | | | | NME_0964 | | | | NMW_0979 | NLA1270 |
| gna1870 | Factor H-binding protein | | X | | | | | | NMO0296 | | | NMA0586 | | | | | NMB1870 | | | | | NMC0349 | | | | NME_1556 | | | | NMW_0880 | - |
| gna1946 | Putative lipoprotein | | P | | | | | | NMO0225 | | | NMA0506 | | | | | NMB1946 | | | | | NMC1917 | | | | NME_1556 | | | | NMW_2495 | NLA1270 |
| gna2132 | Putative lactoferrin-binding protein | | X | | | | | | NMO0042 | | | NMA0299 | | | | | NMB2132 | | | | | NMC2109 | | | | NME_1876 | | | | NMW_2154 | NLA20270 |
| hap | IgA-specific serine endopeptidase | | M, U | | | | | | NMO0193 | | | NMA0457 | | | | | NMB1985 | | | | | NMC1959 | | | | NME_2246 | | | | NMW_1993 | NLA19200 |
| HaemH | Ferrochelatase | | H | | | | | | NMO0608 | | | NMA0927 | | | | | NMB0718 | | | | | NMC0669 | | | | NME_0263 | | | | NMW_1872 | NLA15160 |
| HaemH | Phosphoribosylaminoimidazole-succinocarboxamide synthase | | F | | | | | | NMO0646 | | | NMA0968 | | | | | NMB0757 | | | | | NMC0709 | | | | NME_0288 | | | | NMW_0033 | NLA14730 |
| misR/phoP | Putative two-component system response regulator | | T, K | | | | | | NMO0480 | | | NMA0798 | | | | | NMB0595 | | | | | NMC0537 | | | | NME_1391 | | | | NMW_0132 | NLA16380 |
| misS/phoQ | Sensor histidine kinase | | T | | | | | | NMO0479 | | | NMA0797 | | | | | NMB0594 | | | | | NMC0536 | | | | NME_1392 | | | | NMW_0133 | NLA16390 |
| mtrC | Membrane fusion protein | | M | | | | | | NMO1532 | | | NMA1970 | | | | | NMB1716 | | | | | NMC1634 | | | | NME_0708 | | | | NMW_2191 | NLA5560 |
| mtrD | Putative efflux system transmembrane protein | | V | | | | | | NMO1531 | | | NMA1969 | | | | | NMB1715 | | | | | NMC1633 | | | | NME_0709 | | | | NMW_2192 | NLA5570 |
| mtrE | Probable outer membrane lipoprotein | | M, U | | | | | | NMO1530 | | | NMA1968 | | | | | NMB1714 | | | | | NMC1632 | | | | NME_0710 | | | | NMW_2193 | NLA5580 |
| mtrR | Transcriptional regulator mtrR | | K | | | | | | NMO1533 | | | NMA1971 | | | | | NMB1717 | | | | | NMC1635 | | | | NME_0707 | | | | NMW_2190 | NLA5550 |
| narE | Hypothetical protein NMB1343 | | - | | | | | | - | | | - | | | | | NMB1343 | | | | | - | | | | - | | | | - | - |
| natC | Putative outer membrane protein | | M, U | | | | | | NMO1555 | | | NMA1994 | | | | | NMB1737 | | | | | NMC1657 | | | | NME_1147 | | | | NMW_0991 | - |
| nlpD | Putative membrane peptidase | | M | | | | | | NMO1312 | | | NMA1692 | | | | | NMB1483 | | | | | NMC1418 | | | | NME_2339 | | | | NMW_2516 | - |
| norZ | Nitric-oxide reductase | | P | | | | | | NMO1451 | | | NMA1886 | | | | | NMB1622 | | | | | NMC1548 | | | | NME_0084 | | | | NMW_0421 | NLA6500 |
| nspA | Surface antigen | | M | | | | | | NMO0549 | | | NMA0862 | | | | | NMB0663 | | | | | NMC0612 | | | | NME_0907 | | | | NMW_1906 | NLA15790 |
| nth | Endonuclease III | | L | | | | | | NMO0415 | | | NMA0711 | | | | | NMB0533 | | | | | NMC0472 | | | | NME_0533 | | | | NMW_2251 | NLA17060 |
| prc | Putative carboxyl-terminal processing protease | | M | | | | | | NMO1172 | | | NMA1546 | | | | | NMB1332 | | | | | NMC1270 | | | | NME_0500 | | | | NMW_0298 | NLA11670 |
| vacJ | Putative lipoprotein | | M | | | | | | NMO0210 | | | NMA0490 | | | | | NMB1961 | | | | | NMC1933 | | | | NME_1405 | | | | NMW_1203 | NLA19040 |
| vapA | Aida-related Type V secretory pathway adhesin | | M, U | | | | | | NMO1728 | | | NMA2175 | | | | | - | | | | | NMC1859* | | | | NME_0521 | | | | NMW_2310 | NLA3700 |
| virG | Putative autotransporter protein | | M, U | | | | | | NMO1346 | | | NMA1725 | | | | | NMB1525* | | | | | NMC1454* | | | | NME_1269 | | | | NMW_0558 | - |

^†^ For α 153 and α 275 the absence of a putative orthologous gene may be due to either missing data or missing annotation as for both strains only draft genome sequences were obtained which were annotated automatically (Schoen et al, PMID: 18305155).

* Putative pseudogene.

This table was produced by adding the *N. lactamica* 020-06 data to the table created by Schoen et al (PMID: 18305155).
